# Mid‐Infrared on‐Chip Dichroic Filters Enabled by Adiabatic Evolution and Dispersion‐Engineered Subwavelength Grating Waveguides

**DOI:** 10.1002/nap2.70279

**Published:** 2026-08-29

**Authors:** Yuheng Liu, Wenchen Yin, Yuhan Sun, Lipeng Xia, Yulin Wu, Xiaochuan Xu, Yi Zou

**Affiliations:** ^1^ School of Information Science and Technology ShanghaiTech University Shanghai China; ^2^ State Key Laboratory on Tunable Laser Technology Harbin Institute of Technology Xili University Town Harbin Institute of Technology Campus Shenzhen Guangdong China; ^3^ State Key Laboratory of Materials for Integrated Circuits Shanghai Institute of Microsystem and Information Technology, Chinese Academy of Sciences Shanghai China

**Keywords:** dichroic filter, mid‐infrared, silicon photonics, subwavelength grating

## Abstract

Broadband high‐performance optical filters are fundamental building blocks for advancing mid‐infrared (MIR) integrated photonics, particularly for applications in molecular sensing and free‐space communication. Although dichroic filters offer robust high‐roll‐off performance in the near‐infrared, their realization in the critical MIR atmospheric transparency windows (3–5 μm) has remained largely unexplored. In this work, we demonstrate a high‐performance on‐chip dichroic filter on a silicon‐on‐insulator (SOI) platform, specifically engineered for the 3300–3700 nm spectral range. By integrating a longitudinally segmented subwavelength grating (SWG) waveguide, we effectively mitigate slot‐like propagation losses induced by sidewall roughness while simultaneously achieving precise control over mode dispersion. Through the synergistic adiabatic evolution and asymmetric directional coupler theory, the device achieves a maximum roll‐off rate of approximately 1 dB/nm and an extinction ratio exceeding 12 dB at a measured cut‐off wavelength of *λ*_c_ ≈ 3460 nm. The filters exhibit flat‐top low‐pass and high‐pass characteristics over a 400‐nm bandwidth with negligible insertion loss. Our results demonstrate strong agreement between theoretical analysis and experimental validation, providing a scalable solution for interchannel crosstalk suppression and high‐SNR signal extraction in MIR photonic integrated circuits. This work fills a critical gap in the MIR toolkit, paving the way for sophisticated on‐chip spectroscopic systems and robust free‐space optical links.

## Graphical abstract

An on‐chip mid‐infrared dichroic filter is demonstrated using an asymmetric directional coupler theory comprising a longitudinally segmented subwavelength‐grating waveguide and a strip waveguide. Suppressed slot‐like scattering loss and precise dispersion engineering yield flat‐top responses over 3.3–3.7 μm, ∼0.9 dB insertion loss, ∼1 dB/nm roll‐off, and > 12 dB extinction ratio for scalable integrated spectroscopy and free‐space optical links.

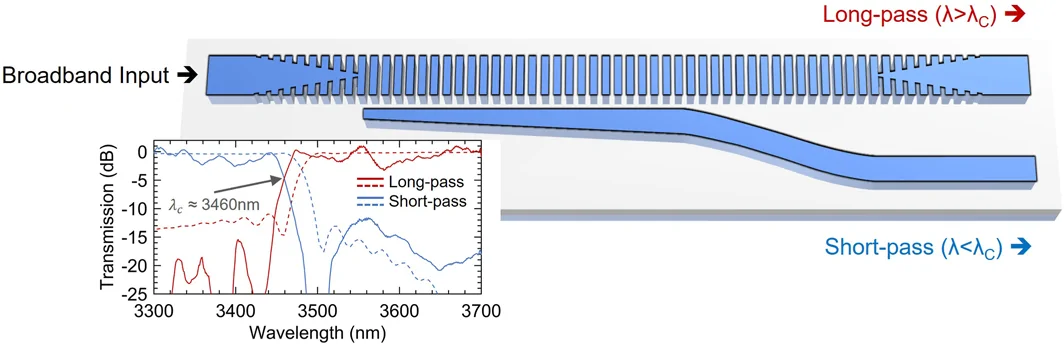

## 1 Introduction

The mid‐infrared (MIR) spectral region (2–20 μm) has emerged as a rapidly growing domain in integrated photonics due to its unique optical properties and broad application potential in environmental monitoring [1], industrial sensing [2], and biomedicine [3]. Compared with the near‐infrared (NIR) regime, the MIR offers distinct advantages in both light propagation and light–matter interaction, making it particularly attractive for next‐generation photonic systems [1, 4, 5].

From a propagation perspective, MIR wavelengths experience reduced scattering loss under adverse atmospheric conditions, such as haze and aerosols, due to the weaker Mie scattering at longer wavelengths [6]. In addition, MIR signals exhibit improved resilience against atmospheric turbulence, including phase distortion and intensity scintillation, enabling more stable transmission in free‐space optical links [7]. These characteristics make the MIR region highly advantageous for robust optical communication in complex environments.

From a sensing standpoint, the MIR encompasses two critical atmospheric transparency windows (3–5 and 8–12 μm), which overlap with the fundamental vibrational absorption bands of most molecules [1, 8]. This leads to light–matter interactions that are typically an order of magnitude stronger than those in the NIR, establishing the MIR as the so‐called “molecular fingerprint” region. As a result, MIR photonic systems enable label‐free, in situ sensing with high selectivity and detection limits down to the parts‐per‐billion (ppb) level, while remaining inherently immune to electromagnetic interference.

To fully exploit these advantages, broadband optical filters with high roll‐off rates are essential components in MIR systems [9]. In integrated photonic circuits, such filters suppress interchannel crosstalk, thereby improving spectral efficiency and signal integrity. In free‐space or off‐chip configurations, they are critical for rejecting background radiation and enhancing the signal‐to‐noise ratio (SNR). In spectroscopic sensing, broadband filters enable accurate extraction of target absorption features from complex hyperspectral backgrounds, directly impacting measurement sensitivity and reliability.

A variety of on‐chip filtering approaches have been proposed to date [10, 11, 12, 13, 14, 15, 16]. Among them, filters based on adiabatic directional coupler (DC) theory are particularly promising as they can simultaneously achieve broadband operation and sharp spectral roll‐off [10, 11, 12]. However, existing demonstrations have been largely restricted to the NIR regime (as summarized in Table 1), leaving the MIR region comparatively underexplored. This limitation is primarily attributed to the increased testing difficulty and optical loss in MIR‐compatible waveguide platforms, which have hindered the realization of high‐performance adiabatic devices.

**TABLE 1:** Summary of the advances in adiabatic filters based on asymmetric DC theory.

Cut‐off wavelength (μm)	Coupling length (μm)	Total length (μm)	Roll‐off rate (dB/nm)	ER (dB)	Insertion loss (dB)	Ref.
1.54	260	1560	2.82	∼10	< 2	[10]
2.1	350	1850	0.64	∼15	< 2	[10]
1.53	N.A.	766.8	1.4	14.5	< 1.5	[11]
1.63	N.A.	1126.4	1.55	11	< 1.5	[11]
1.54	50	240	N.A.	∼12	0.8	[12]
3.46	274.5	385	∼1.0	12	∼0.9	This work

*Note:* See Supporting Information S1: Note 1 for more detailed discussion on a wavelength‐independent roll‐off rate for cross‐band comparison.

In this work, we address this gap by demonstrating a dichroic filter operating in the 3–5 μm atmospheric transparency window based on asymmetric DC theory. The device is implemented on a CMOS compatible and scalable silicon‐on‐insulator (SOI) platform. To avoid the strongly slot‐like modal distribution typically observed in transversely segmented SWG or slot‐like waveguides, which leads to increased optical‐mode overlap with etched sidewall interfaces and hence greater sensitivity to sidewall roughness, a longitudinally segmented subwavelength grating (SWG) waveguide is used in place of conventional transverse segmentation [17]. Experimental results reveal a maximum roll‐off rate of ∼1 dB/nm and an extinction ratio (ER) exceeding 12 dB. The measured performance agrees well with theoretical predictions and numerical simulations, validating the proposed design and offering a powerful tool for MIR spectroscopic and communication systems.

## 2 Design and Fabrication

The proposed high‐roll‐off dichroic filter is architected as an asymmetric coupled‐waveguide system, comprising a fixed‐width SWG waveguide and an adjacent strip waveguide with a spatially evolving width (Figure 1a). Transverse electric (TE) polarized light is launched into the initial strip section and smoothly transitioned into the guided mode of the SWG architecture via a tailored strip‐to‐SWG converter [18]. The spectral separation mechanism relies on wavelength‐dependent modal evolution: spectral components shorter than the critical cut‐off wavelength (λ < $\lambda_{c}$) are adiabatically transferred to the lower strip waveguide, whereas longer‐wavelength components (λ > $\lambda_{c}$) remain localized within the SWG backbone and are subsequently recovered into a conventional strip mode. The geometric footprint is defined by the converter length ($L_{C}$ = 18 μm), the adiabatic coupling region ($L_{\text{CR}}$ = 274.5 μm), and the mode separation region ($L_{\text{SR}}$ = 74.5 μm). Detailed structural parameters (Figure 1b) are summarized in Table 2.

**FIGURE 1:**
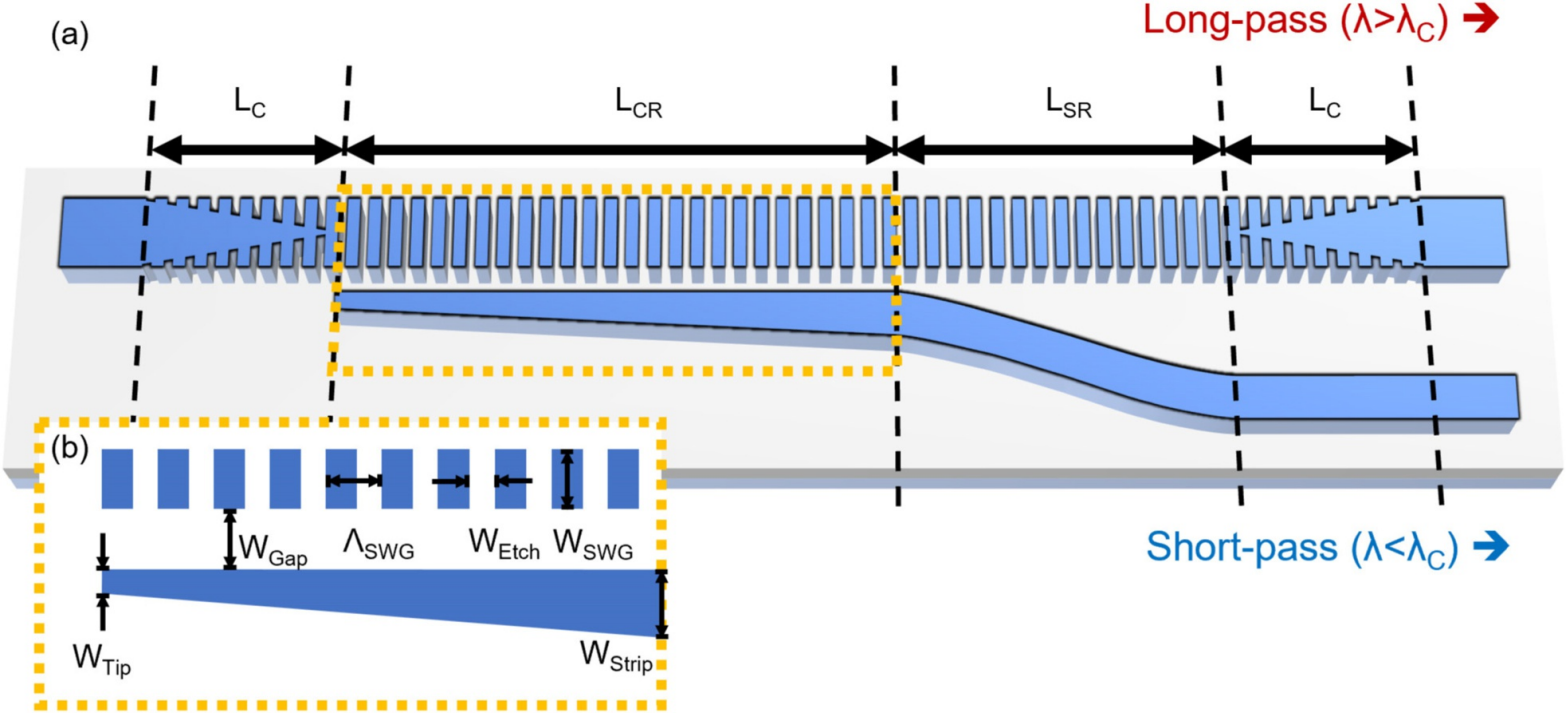
Architectural framework of the proposed mid‐infrared dichroic filter. (a) Schematic of the proposed dichroic filter operating based on asymmetric directional coupler (DC) theory. (b) Magnified top‐view schematic detailing the coupling region, highlighting the smooth transition profiles of the strip and SWG waveguides.

**TABLE 2:** Optimized geometric parameters of the proposed MIR dichroic filter.

$L_{C}$	$L_{\text{CR}}$	$L_{\text{SR}}$	$W_{\text{Tip}}$	$W_{\text{Strip}}$	$\Lambda_{\text{SWG}}$	$W_{\text{Etch}}$	$W_{\text{SWG}}$	$W_{\text{Gap}}$
18	274.5	74.5	0.2	0.8	0.45	0.2	2.6	0.2

*Note:* Unit: μm.

Our design framework is underpinned by Asymmetric DC theory. For a system of two dissimilar waveguides (denoted as WG_A_ and WG_B_), the supermode propagation constants (β) and their corresponding field distributions are governed by the following eigenvalue equation [10, 19]:

(1) $$\left[\begin{array}{cc} \beta_{A} & \kappa \\ \kappa^{*} & \beta_{B} \end{array}\right]\psi =\beta \psi ,$$

where $\beta_{A}$ and $\beta_{B}$ represent the intrinsic propagation constants of the isolated waveguides, and κ denotes the cross‐coupling coefficient. To suppress parasitic mode‐hopping and ensure high‐fidelity spectral routing, the system must satisfy the adiabatic criterion, where geometric variations are sufficiently gradual to allow the optical power to continuously track the instantaneous fundamental quasi‐even supermode [20]. For the two‐supermode system presented in our work, this condition can be expressed as follows [21]:

(2) $$\left|\langle \psi_{-}\left(x\right)\left|\frac{d}{dz}\right|\psi_{+}\left(x\right)\rangle \right|\ll |\beta_{+}\left(x\right)-\beta_{-}\left(x\right)|,$$

where $\psi_{+}\left(x\right)$ and $\psi_{-}\left(x\right)$ denote the quasi‐even and quasi‐odd supermodes, respectively. $\beta_{+}\left(x\right)$ and $\beta_{-}\left(x\right)$ are their corresponding propagation constants. In the proposed dichroic filter, the relevant adiabatic eigenmode is its fundamental supermode (the quasi‐even mode). The normalized expression can be denoted as $\psi_{+}=\frac{1}{\sqrt{2}}\left[\sqrt{1+\delta /\sqrt{\delta^{2}+{\left|\kappa \right|}^{2}}};\;\sqrt{1-\delta /\sqrt{\delta^{2}+{\left|\kappa \right|}^{2}}}\right]$, where $\delta =\left(\beta_{A}-\beta_{B}\right)/2$ represents the phase mismatch. The spatial power distribution at the device termini is quantified by the power ratios $r_{T}$:

(3) $$r_{T}=\frac{\sqrt{\delta^{2}+{\left|\kappa \right|}^{2}}+\delta }{\sqrt{\delta^{2}+{\left|\kappa \right|}^{2}}-\delta }.$$

For a given pair of waveguides, the isolated propagation constants $\beta_{A}$ and $\beta_{B}$ are fixed at each wavelength, so the phase mismatch δ is uniquely determined. Taking the isolated SWG waveguide and strip waveguide used in this work as an example, the corresponding dependence of $r_{T}$ on κ is plotted in Figure 2c. At the cut‐off wavelength ($\lambda_{c}$ ≈ 3485 nm in simulation), the phase mismatch condition leads to $r_{T}=0\;\text{dB}$, indicating an equitable power split between the two channels. As λ deviates from $\lambda_{c}$, the rapid divergence of the phase mismatch δ drives a swift redistribution of the modal field as indicated by the rapidly increased magnitude of $\left|r_{T}\right|$. Specifically, for $\lambda \;<\lambda_{c}$ (δ<0 and $r_{T}\;<\;0$), the higher effective index ($n_{\text{eff}}$) of the lower strip waveguide traps the majority of the power. Conversely, for $\lambda \;>\lambda_{c}$ ($r_{T}\;>\;0$), the SWG waveguide dominates the modal confinement due to its engineered dispersion profile (Figure 2a,b,d,e).

**FIGURE 2:**
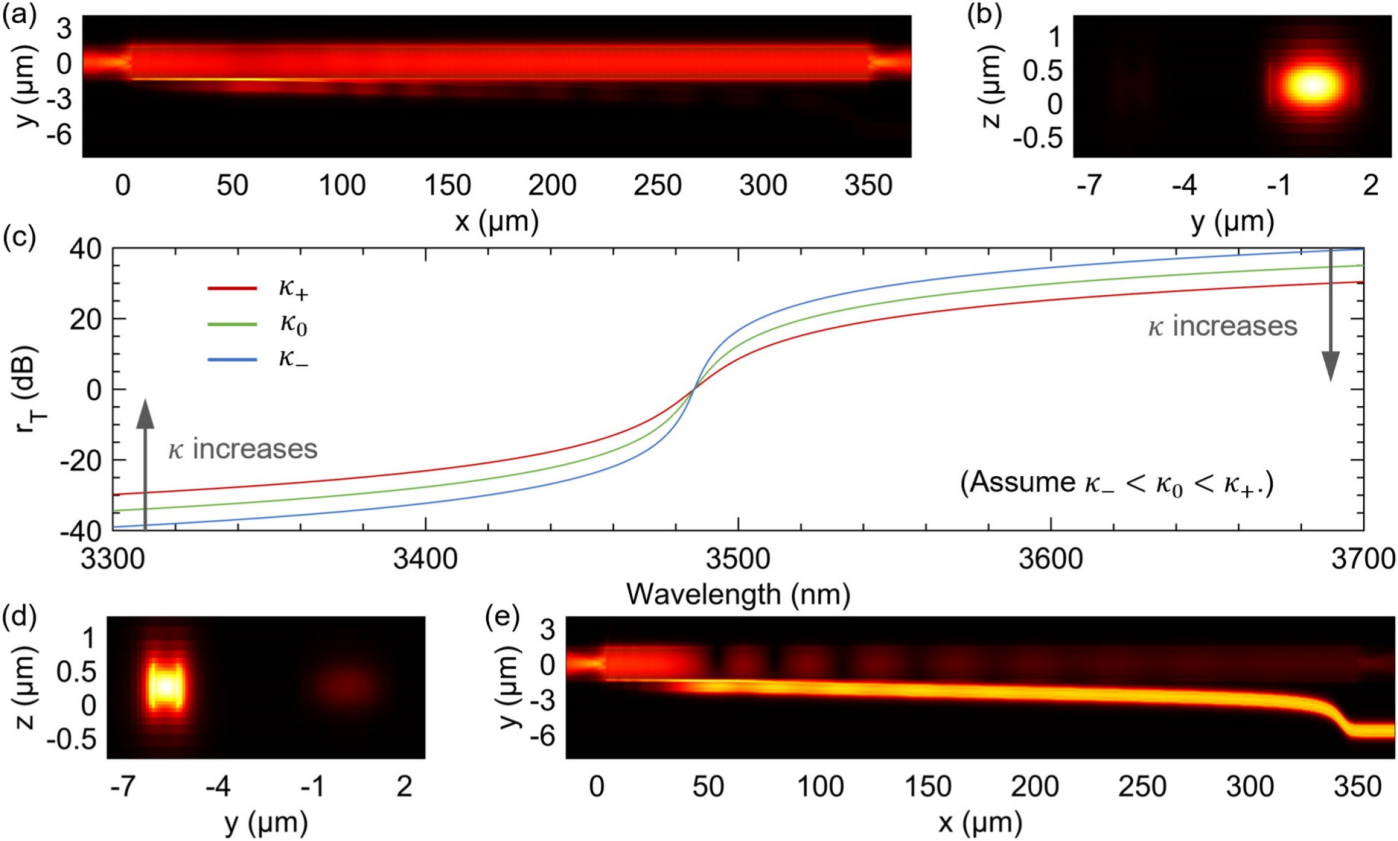
Theoretical analysis and numerical simulation of the adiabatic mode evolution. (a) Simulated light propagation profiles showing the optical field distribution when the operational wavelength λ > $\lambda_{c}$, and (b) the corresponding cross‐sectional mode profile at the output port. (c) Theoretically calculated power ratio ($r_{T}$) at the device termini as a function of wavelength for various coupling coefficients (κ). A smaller κ yields a steeper spectral roll‐off rate. (d) Simulated light propagation profiles when λ < $\lambda_{c}$ and (e) the corresponding cross‐sectional mode profile, demonstrating highly efficient spatial mode routing.

This wavelength‐dependent power redistribution facilitates efficient spectral bifurcation, enabling the routing of specific components to designated output ports relative to the cut‐off wavelength $\lambda_{c}$. Analytically, a reduction in the coupling coefficient κ yields a steeper $r_{T}$ response near the transition point, directly translating to a sharper filter roll‐off. However, this performance gain involves a critical trade‐off: a smaller κ necessitates a prolonged interaction length to ensure complete adiabatic power transfer. Given that our device is implemented on an SOI platform, its buried oxide layer begins to exhibit noticeable absorption beyond ∼3.6 μm^4^ (See Supporting Information S1: Note 2 for more detailed discussion on MIR material platforms). Therefore, although a longer device theoretically provides a steeper roll‐off, the insertion loss of the fabricated device would also increase in the long‐wavelength part of the measurement range, particularly around 3600–3700 nm that we intend to characterize. To ensure the practicality of our design for future work, it is essential to moderately sacrifice the roll‐off rate, that is, shorten the coupling length, in order to reduce insertion loss and minimize the device footprint. This constitutes the first key trade‐off in our design: the trade‐off between roll‐off rate and insertion loss.

In our architectural implementation, the long‐pass port utilizes an SWG waveguide to induce a dispersion profile distinct from that of the conventional strip waveguide. A wide range of geometrical parameters for both strip and SWG waveguides were explored to achieve the desired filtering performance as shown in Figure 3a. The SWG waveguide exhibits an $n_{\text{eff}}$ dispersion that differs from that of a conventional strip waveguide. This distinct dispersion enables the two uncoupled waveguides to satisfy the phase‐matching condition at a specific wavelength within the operating band, thereby defining the theoretical cut‐off wavelength of the dichroic filter. In practice, the $n_{\text{eff}}$ dispersion of the SWG waveguide can be flexibly tailored by adjusting its width and FF. Such dispersion engineering introduces a wavelength‐dependent phase mismatch between the SWG and strip waveguides, which is the fundamental mechanism underlying the dichroic filtering functionality. Specifically, the dispersion profiles of the SWG and strip waveguides are designed to intersect at a predetermined wavelength, corresponding to the cut‐off wavelength of the filter. Consequently, by varying the SWG width and FF, the position of this intersection and hence the cut‐off wavelength can be precisely shifted to meet different application requirements. Specifically, to ensure the SWG functions as a homogeneous effective medium and suppresses unwanted Bragg reflections or diffraction, the grating period $\Lambda_{\text{SWG}}$ is strictly maintained within the deep‐subwavelength regime ($\Lambda_{\text{SWG}}\ll \lambda /{2n}_{\text{eff}}$) [22].

**FIGURE 3:**
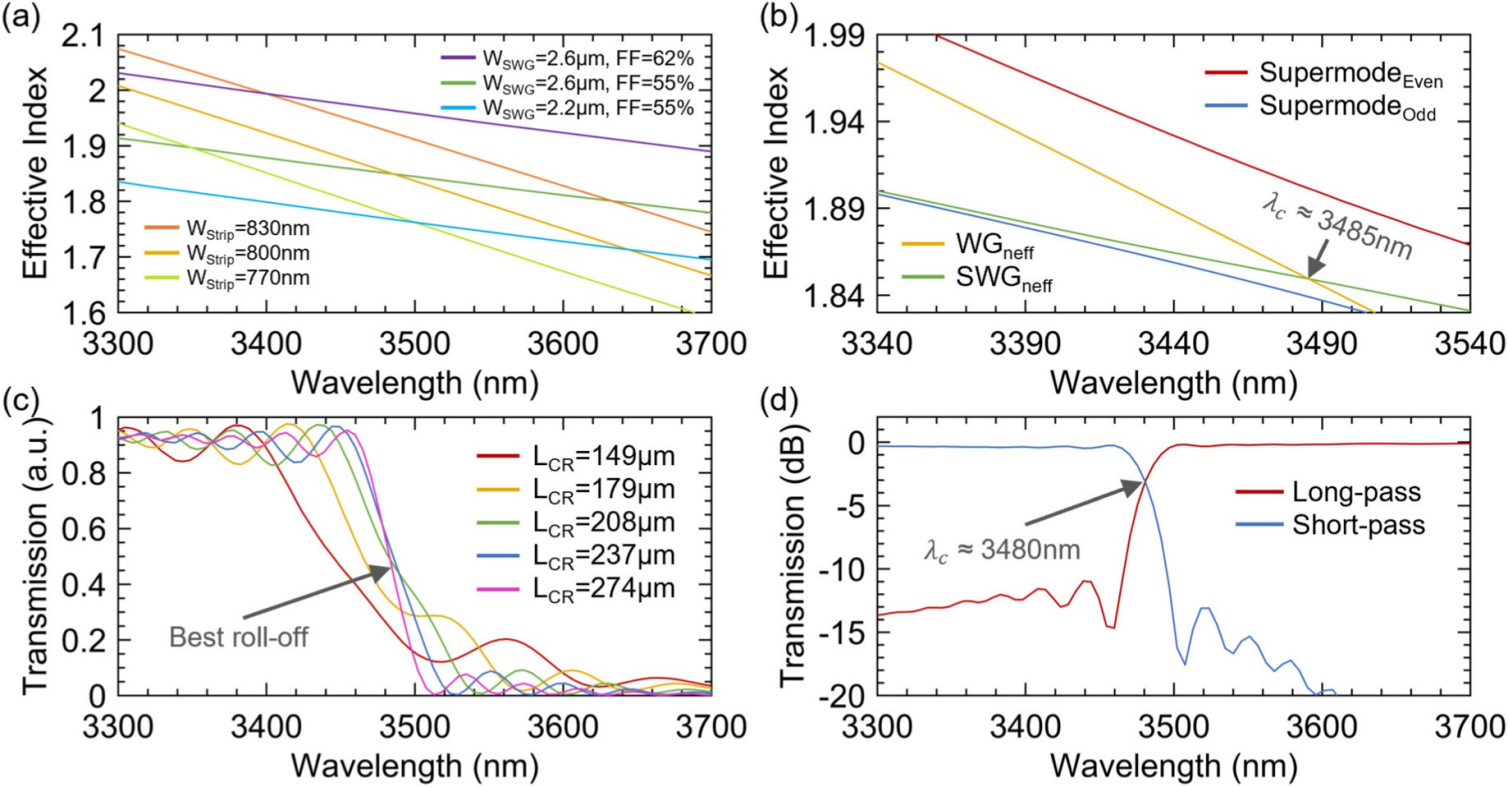
Dispersion engineering, geometric optimization, and simulated spectral responses. (a) Calculated effective indices ($n_{\text{eff}}$) of the isolated strip waveguides and SWG waveguides across a broad range of geometric parameters. (b) Optimized waveguide dimensions implemented in this work alongside the calculated indices of the supported quasi‐even and quasi‐odd fundamental supermodes. (c) Simulated transmission spectra at the short‐pass port under different adiabatic coupling lengths ($L_{\text{CR}}$), illustrating the optimization process. (d) Full‐wave simulated dual‐port spectral response of the optimized dichroic filter.

Similarly, as shown in Figure 3a, several combinations of geometric parameters for the SWG and strip waveguides can be selected to achieve a cut‐off wavelength of approximately 3500 nm. When making the final selection, in addition to the aforementioned trade‐off between roll‐off performance and insertion loss, fabrication feasibility and robustness must also be taken into account. Specifically, a trade‐off must be made between the FF of the SWG waveguide and the tolerance of the lithographic process. From a fabrication perspective, the design is carefully tailored to the sensitivities of positive‐resist lithography. We optimized the filling factor (FF) to approximately 55% to navigate the delicate balance between feature broadening under overexposure and incomplete development under underexposure, while mitigating proximity effects in dense patterns. This FF, combined with a period of 450 nm, satisfies the process's robust etch limit of ∼200 nm. Furthermore, the longitudinal segmentation of the SWG architecture was adopted to reduce the slot‐like field concentration near etched sidewalls and the associated mode overlap with rough dielectric interfaces. Compared with transversely segmented SWG or slot‐like waveguides, where the TE‐like modal energy can be strongly concentrated in low‐index gaps and near rough Si interfaces, the longitudinally segmented SWG preserves a laterally continuous silicon region and retains more modal energy inside the high‐index core. This mode distribution is theoretically expected to reduce the sensitivity to sidewall‐roughness‐induced scattering. To further enhance robustness, the SWG width is chosen to be 2.6 μm, a regime where the effective index $n_{\text{eff}}$ becomes less sensitive to width variations (See Supporting Information S1: Note 3 for more detailed information). However, increasing the width beyond this point would result in a longer required coupling length. Considering the constraints of the experimental setup, the maximum width of the strip waveguide in the coupling region is limited to 0.8 μm. In other words, by selecting appropriate waveguide widths, we achieve a balance among the roll‐off rate, insertion loss, and fabrication robustness. Increasing the widths of both the SWG and strip waveguides reduces the sensitivity of the $n_{\text{eff}}$ to waveguide‐width variations, thereby improving agreement between the theoretical design and experimental results. It also suppresses sidewall‐scattering loss, reduces device insertion loss, and weakens coupling between the waveguides, further improving the roll‐off rate. However, wider waveguides require a longer coupling length to achieve sufficient adiabatic power transfer, which may in turn increase absorption loss in the long‐wavelength region. The final device parameters, as listed in Table 2, ensure a favorable roll‐off rate and low insertion loss while also accounting for lithographic process tolerance and fabrication robustness. The corresponding quasi‐even and quasi‐odd supermodes at the end of the adiabatic evolution are shown in Figure 3b. Mutual coupling lifts the mode degeneracy, leading to supermode splitting at $\lambda_{c}$. Finally, the coupling region contours are defined by Bezier curves. This smooth geometric transition eliminates abrupt curvature changes, thereby minimizing parasitic mode mismatch and ensuring that the system remains adiabatically confined to the fundamental quasi‐even and quasi‐odd supermodes throughout the evolution.

The adiabatic evolution length was optimized using a numerical approach based on efficient “adiabatic mode evolution structures” [20]. Taking the lower strip waveguide (i.e., the short‐pass port) as an example, increasing the coupling length enhances adiabaticity, which is manifested as a steeper spectral roll‐off. However, this improvement becomes marginal once the length reaches approximately 274.5 μm as shown in Figure 3c. At this optimized length, the dual‐port transmission spectra of the proposed filter are presented in Figure 3d. At the cutoff wavelength $\lambda_{c}$ ≈ 3480 nm, the transmission spectra of the two output ports intersect at approximately −3 dB. Compared with the theoretical prediction, the simulated $\lambda_{c}$ exhibits a slight shift. This discrepancy is expected as the evanescent field distribution of the SWG waveguide in the full‐wave simulation differs from that of the equivalent homogeneous waveguide assumed in the theoretical model. Additional minor deviations may also arise from the finite mesh resolution used in the simulations.

The mode separation region is placed after the main adiabatic coupling region. Its function is to spatially separate the two waveguides after the wavelength‐dependent modal evolution has occurred, thereby reducing residual coupling between the output channels and allowing the two modes to be routed to their respective output ports. The separation region was designed as a Bézier‐curve‐based waveguide transition. Several control points were defined along the propagation direction to describe the gradual fan‐out of the two waveguides. The total length and the control‐point positions were then optimized numerically using a particle swarm optimization algorithm. The figure of merit included the transmission performance of the target output channels together with a compactness constraint. Based on this optimization, the final longitudinal length of the mode separation region was chosen to be $L_{\text{SR}}$ = 74.5 μm.

## 3 Results and Discussion

The proposed MIR dichroic filters were fabricated on an SOI platform with a 500‐nm‐thick top silicon layer and a 3‐μm‐thick buried oxide (BOX) layer, and the detailed fabrication processes is described in ref. [23]. As depicted in the optical image (Figure 4a) and scanning electron microscopy (SEM) images (Figure 4b–d), the structural integrity of the SWG and the adiabatic taper regions is well‐maintained. The measured dimensions of the SWG segments and coupling gaps are in good agreement with the design, with deviations within typical fabrication tolerances, confirming the robustness and high fidelity of the nanofabrication process.

**FIGURE 4:**
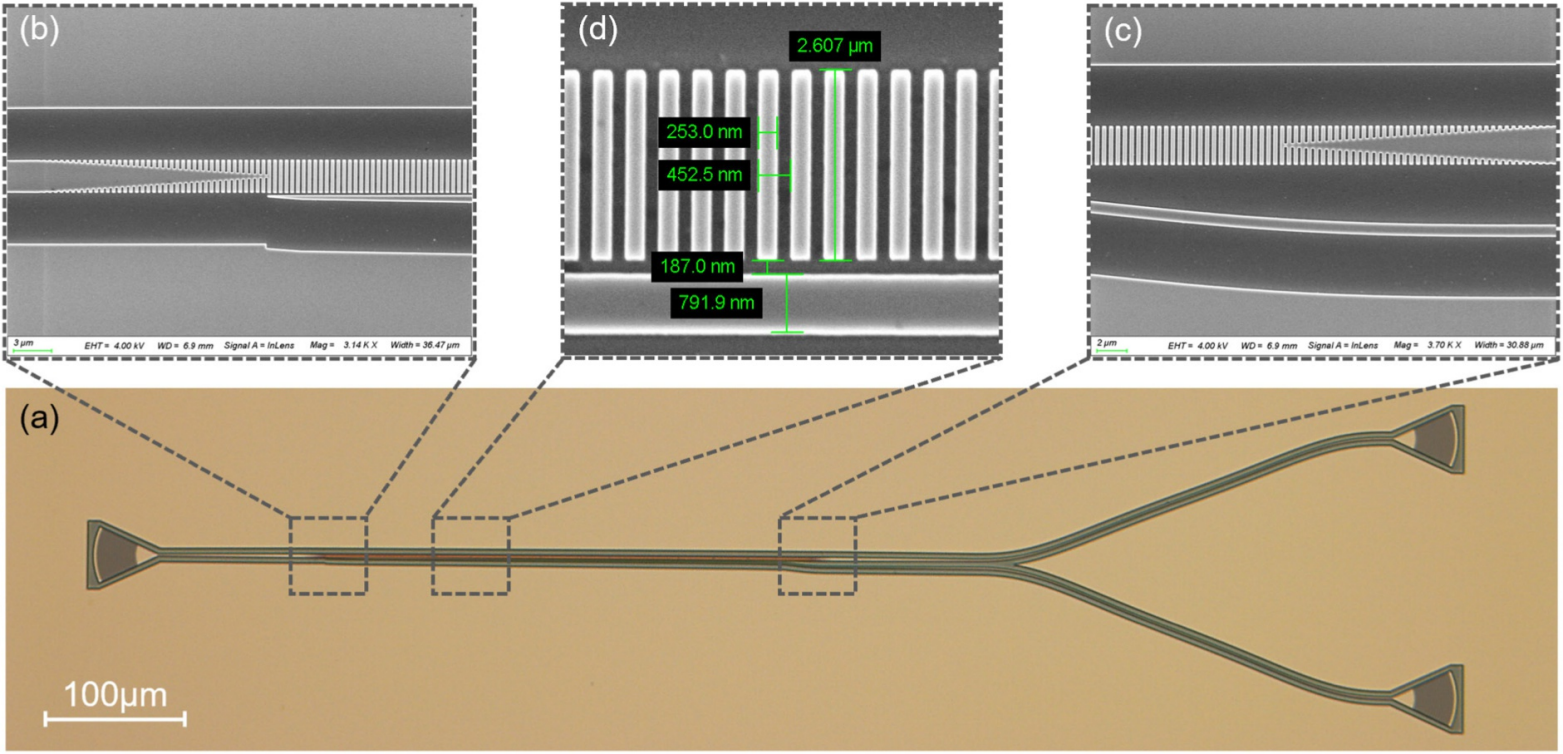
| Morphological characterization of the fabricated device. (a) Optical micrograph of the fully integrated MIR dichroic filter. (b–d) Representative SEM images captured at different functional regions of the device, confirming high‐fidelity nanofabrication and structural integrity of the subwavelength grating segments.

For optical characterization, MIR grating couplers [24] were used for fiber‐to‐chip coupling, enabling efficient coupling of off‐chip light into the chip. Mode adapters were placed after the grating couplers to suppress higher‐order mode excitation and improve coupling to the single‐mode access waveguides. In the strip‐to‐SWG converter, the central taper length was designed to be 18 μm to ensure nearly lossless adiabatic mode conversion. Experimentally, back‐to‐back strip‐SWG‐strip test structures incorporating the same converters did not exhibit a pronounced excess loss compared with standard strip waveguides of the same total length (See Supporting Information S1: Note 9 for more details).

To control the input polarization, a half‐wave plate (Thorlabs WPLH05M‐3500) was employed to compensate for polarization fluctuations introduced by nonpolarization‐maintaining MIR fibers, thereby maximizing the coupling efficiency with the high polarization ER of the grating couplers. The normalized dual‐port transmission spectra of the fabricated device are shown in Figure 5, where the wavelength‐dependent response of the input/output grating couplers was removed using reference structures fabricated on the same chip. Over the full tuning range of the laser source (3300–3700 nm), the device demonstrates both low‐pass and high‐pass filtering enabled by adiabatic mode evolution. Broadband flat‐top responses are observed, with passband widths exceeding 150 nm for the low‐pass port and 250 nm for the high‐pass port. The ER at both output ports exceeds 12 dB. Near the cut‐off wavelength ($\lambda_{c}$ ≈ 3460 nm), the device exhibits a maximum roll‐off rate of ∼1 dB/nm and an average roll‐off rate of ∼0.8 dB/nm, indicating highly efficient modal discrimination (See Supporting Information S1: Notes 4 and 5 for more discussion on roll‐off rate and ER). It is also worth noticing that the BOX absorption is not clearly observed in the normalized long‐pass transmission spectrum because the total length of the proposed filter is relatively short (∼385 μm).

**FIGURE 5:**
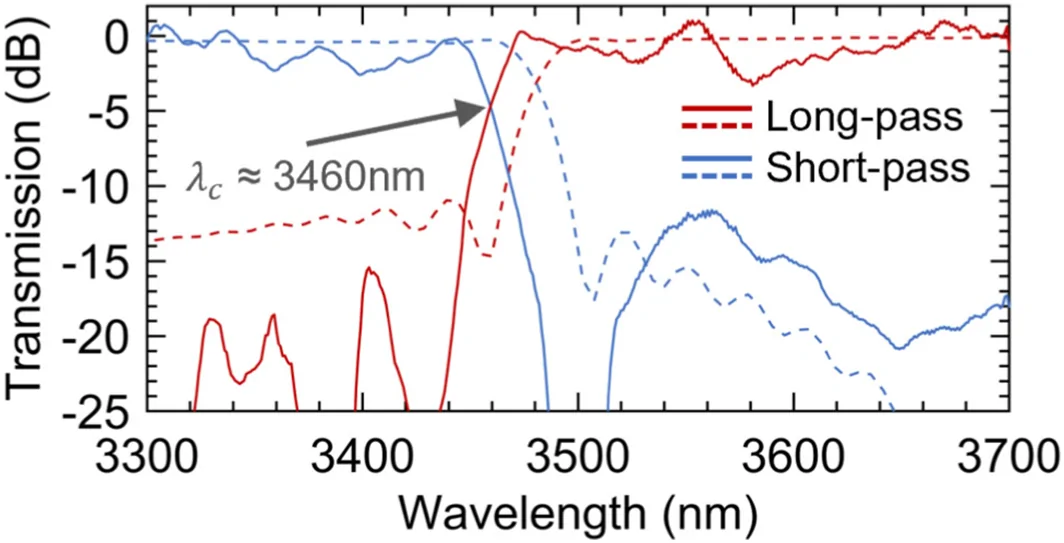
Experimentally measured spectral performance of the fabricated MIR dichroic filter. Dual‐port transmission spectra of the fabricated device, characterized over the full tuning range of the laser source (3300–3700 nm). Solid lines represent the experimentally measured results, whereas dashed lines denote the corresponding simulation data, exhibiting an excellent agreement in their spectral trends. The cut‐off wavelength is highlighted by the black arrow at $\lambda_{c}$ ≈ 3460 nm. The device demonstrates broadband flat‐top low‐pass and high‐pass filtering enabled by adiabatic mode evolution, with an ER exceeding 12 dB at both output ports and a maximum roll‐off rate of 1 dB/nm near the cut‐off point. A part of transmission values slightly above 0 dB arise from the normalization procedure and measurement uncertainty. Since the spectra of the dichroic filter are normalized to the reference grating structures which were measured separately, small differences in grating‐coupler efficiency, fiber alignment, and polarization state can lead to normalized values slightly exceeding 0 dB, which does not indicate optical gain (See Supporting Information S1: Note 8 for more discussion).

Compared with the theoretical prediction ($\lambda_{c}$ ≈ 3480 nm), a slight blue shift was observed in the experimental results. This discrepancy is primarily attributed to fabrication‐induced variations. SEM analysis Figure 4d shows that the width deviations of both the SWG and strip waveguides are within ∼15 nm, whereas the filling factor (FF) increases by less than ∼4%. Although these deviations are within typical fabrication tolerances, they induce subtle changes in the effective indices and dispersion profiles, leading to the observed shift in the cutoff wavelength (See Supporting Information S1: Notes 6 and 7 for more detailed discussion). Nonetheless, the measured results are in excellent agreement with full‐wave simulations, confirming the validity and reliability of the design.

The demonstrated performance highlights the effectiveness of combining asymmetric DC theory with adiabatic mode evolution for MIR filtering. The average insertion loss of the passbands through the filter was measured to be approximately 0.9 dB. This low sub‐1‐dB insertion loss further confirms the advantage of the adiabatic design in minimizing scattering and mode mismatch. Importantly, the cutoff wavelength and passband characteristics can be flexibly engineered by tuning the strip waveguide width or the SWG parameters (e.g., width and filling factor), providing a versatile design space for application‐specific requirements.

## 4 Conclusion

In summary, we have experimentally demonstrated a high‐performance dichroic filter operating in the 3–5 μm wavelength range, effectively addressing the limited availability of such critical devices in the MIR regime. The device achieves broadband flat‐top low‐pass and high‐pass filtering over 3300–3700 nm, featuring an ER exceeding 12 dB and a maximum roll‐off rate of ∼1 dB/nm near the cutoff wavelength. This proposed platform offers excellent scalability and design flexibility. More advanced filtering functionalities, such as ultra‐narrowband or multiband responses, can be realized in the future by cascading multiple filters with tailored cutoff wavelengths. This work provides a highly promising and scalable solution for high‐SNR signal processing in next‐generation MIR on‐chip spectroscopic systems and free‐space optical links.

## Author Contributions

**Yuheng Liu:** writing – original draft, validation, data curation, writing – review and editing, formal analysis, investigation. **Wenchen Yin:** writing – original draft, writing – review and editing, data curation, validation, formal analysis, investigation. **Yuhan Sun:** data curation. **Lipeng Xia:** methodology, software. **Yulin Wu:** methodology. **Xiaochuan Xu:** writing – review & editing, supervision. **Yi Zou:** conceptualization, writing – review and editing, resources, funding acquisition, supervision, project administration.

## Funding

This work was supported by the Open Research Fund of State Key Laboratory of Materials for Integrated Circuits (SKLJC‐K2026‐01), the ShanghaiTech AI Initiative (AI2026B17), and the Key Research and Development Program of Ningxia Hui Autonomous Region (2025BEG01003).

## Conflicts of Interest

The authors declare no conflicts of interest.

## Supporting information


Supporting Information S1

## Data Availability

The data that support the findings of this study are available from the corresponding author upon reasonable request.
