# Supplementary material for: Mid‐Infrared on‐Chip Dichroic Filters Enabled by Adiabatic Evolution and Dispersion‐Engineered Subwavelength Grating Waveguides
Source: Nanophotonics. 2026 Aug 29;15(17):e70279. doi: 10.1002/nap2.70279 (PMC13672136; doi:10.1002/nap2.70279)
Supplement: Supplementary file 1 [file NAP2-15-e70279-s001.docx]

Supplementary Materials for: Mid-infrared On-chip Dichroic Filters Enabled by Adiabatic Evolution and Dispersion-engineered Subwavelength Grating Waveguides

Yuheng Liu,^1^ Wenchen Yin,^1^ Yuhan Sun,^1^ Lipeng Xia,^1^ Yulin Wu,^1^ Xiaochuan Xu,^2^ Yi Zou^1^

^1^ShanghaiTech University, School of Information Science and Technology, Shanghai 201210, China

^2^State Key Laboratory on Tunable Laser Technology, Harbin Institute of Technology, Xili University Town, Harbin Institute of Technology campus, Shenzhen, Guangdong 518055, China

**Correspondence:** Yi Zou (zouyi@shanghaitech.edu.cn) and Xiaochuan Xu (xuxiaochuan@hit.edu.cn)

**Contents**

1. Wavelength-independent Roll-off Rate for Cross-band Comparison

2. Extension of the Proposed Design to Alternative MIR Integrated Photonic Platforms

3. Trade-off between SWG Width and Fabrication Tolerance

4. Discussion on Measured ER

5. Discussion on Measured Roll-off Rate

6. Verification of Fabrication-Induced Cut-off Wavelength Blue Shift

7. Discussion of Thermo-optic Effects on Cut-off Wavelength

8. Interferometric Signatures in the Transmission Spectra

9. Insertion losses of the strip-to-SWG converter

**1	|	Wavelength-independent Roll-off Rate for Cross-band Comparison**

A normalized roll-off rate in units of dB evaluated over 1% relative cut-off wavelength $\lambda_{c}$ will be helpful when comparing roll-off rates in both near-infrared (NIR) and mid-infrared (MIR) regimes. For example, in our device, the measured maximum roll-off is approximately 1 dB/nm near $\lambda_{c}$≈3460 nm. The normalized roll-off rate is therefore:

$$1 dB/nm\times0.01\lambda_{c}\approx34.6dB\left. \right|_{1\%\lambda_{c}}$$

**2	|	Extension of the Proposed Design to Alternative MIR Integrated Photonic Platforms**

The proposed device concept exhibits broadly applicability. We chose the silicon-on-insulator (SOI) platform to demonstrate the proposed device because its low cost and mature CMOS-compatible fabrication ecosystem have made it, and will likely continue to make it, one of the most widely used material platforms in integrated photonics.

Operation slightly beyond this range may still be possible for very short devices or with careful waveguide design, but extending toward 5 μm would likely require an alternative platform.

In recent years, several promising material platforms for MIR integrated photonics have been reported^1–7^. Considering only their material transparency windows and degree of compatibility with CMOS fabrication, potential platforms include Si (1-8.5 μm), Ge (2-14 μm), SiC (0.4-17 μm), Al_2_O_3_ (2-6.6 μm), Ta_2_O_5_ (0.5-10 μm), LiNbO_3_ (0.35-5 μm), Si_3_N_4_ (0.4-6.7 μm), AlN (0.2-13.6 μm), and III-V materials such as InP and InGaAs (approximately 1-10 μm, depending on the specific material composition).

From the perspective of device designing, suspended silicon waveguides, in which the dioxide surrounding the silicon waveguide is removed, also provide a potential route for circumventing the transparency limitation of SiO_2_.

With appropriate redesign, the proposed adiabatic asymmetric-coupler concept and subwavelength grating waveguide (SWG)-based dispersion-engineering strategy could be transferred to these platforms. Nevertheless, compared with conventional silicon platforms or specialized suspended waveguide architectures, many of these alternative platforms still face practical challenges, including relatively low refractive-index contrast, limited compatibility with CMOS fabrication, difficulties in growing sufficiently thick high-quality films, and high fabrication costs.

**3	|	Trade-off between SWG Width and Fabrication Tolerance**

The filter cut-off wavelength is determined by the crossing point between the $n_{eff}$ dispersion curves of the SWG waveguide and the strip waveguide. Therefore, a width-induced perturbation of the SWG dispersion curve can shift the cut-off point. Choosing a relatively wide SWG waveguide is helpful to reduce the sensitivity of the designed cut-off wavelength to fabrication-induced SWG width variations.

As shown in Supplementary Figure 1, the simulated $n_{eff}(\lambda,W_{SWG})$ map shows that $n_{eff}$ increases rapidly for narrow SWG widths but generally enters a flatter region as the width increases. The selected width of $W_{SWG}$ = 2.6 μm lies right within this relatively flat region, where the width sensitivity is reduced. At the same time, further increasing the SWG width would reduce the coupling coefficient between the SWG and strip waveguides and therefore require a longer adiabatic coupling length. Thus, $W_{SWG}$ = 2.6 μm was selected as a compromise between cut-off wavelength robustness, coupling strength, and device footprint.

Further background on SWG waveguides can be found in Ref. [8,9]

**SUPPLEMENTARY FIGURE 1	|**	Simulated $n_{eff}(\lambda,W_{SWG})$ map of longitudinally segmented SWG waveguides as a function of wavelength and SWG width. The red slice shows the dispersion of the SWG waveguide at $W_{SWG}$ = 2.6 μm.

**4	|	Discussion on Measured extinction ratio (ER)**

The extinction ratio (ER) is defined in the conventional way as^10,11^:

$$ER=10\log_{10} \frac{T_{pass}}{T_{filtered}}$$

where $T_{pass}$ is the transmission in the passband and $T_{filtered}$ is the residual transmission at the suppressed output port.

The current ER is mainly limited by practical design and fabrication factors rather than by a fundamental constraint of the asymmetric DC architecture. The main limiting factors include:

1. The adiabatic coupling length was intentionally kept finite to balance roll-off rate, insertion loss and footprint. This finite length leads to residual non-adiabatic leakage and incomplete suppression at the unwanted port.

2. fabrication-induced deviations in the SWG width, filling factor, and coupling gap modify the local effective indices and coupling coefficient, which can shift the designed spectral transition and degrade the port isolation.

3. The measured ER can also be affected by normalization uncertainty and small polarization-state variations in the MIR measurement setup, although these effects were not independently quantified and are not considered the dominant limitation.

4. Weak back reflections from grating couplers and mode converters between straight waveguides and SWG waveguides may also contribute to small spectral ripples, but no strong reflection-induced resonance was observed.

From the perspective of device design alone, higher ER for single proposed filter can be achieved by increasing the adiabatic coupling length, optimizing the longitudinal coupling profile, tightening the fabrication tolerance of the gap and SWG parameters.

The distinctive performance of the proposed dichroic filter lies not only in its high ER, but also in its excellent roll-off rate near the cutoff wavelength. From an engineering perspective, a higher ER can be achieved by cascading multiple proposed filter stages while preserving the sharp roll-off characteristics.

**5	|	Discussion on Measured Roll-off Rate**

The roll-off rate is defined as the slope of the transmission spectrum near the cut-off wavelength. In dB units, it is expressed as^10,11^:

$$Roll-off Rate=\frac{dT_{dB}}{d\lambda}$$

where $T_{dB}$ is the transmission expressed in dB.

The roll-off rate can be improved by increasing the wavelength sensitivity of the mode power redistribution near the cut-off wavelength. In the present design, this can be achieved by reducing the coupling coefficient, increasing the $n_{eff}$ contrast dispersion between the SWG and strip waveguides, and increasing the adiabatic interaction length. Cascading two or more filters with slightly shifted cut-off wavelengths can also significantly improve the effective roll-off rate and stopband rejection.

**6	|	Verification of Fabrication-Induced Cut-off Wavelength Blue Shift**

To verify whether the observed blue shift can be explained by fabrication variations, we re-simulated the $n_{eff}$ dispersion curves of the isolated strip waveguide and SWG waveguide using the actual geometrical parameters extracted from the SEM image shown in Figure 4d. As shown in Supplementary Figure 2, the simulated crossing point between the two dispersion curves shows a cut-off wavelength shift of approximately 23 nm, which agrees well with the experimentally observed blue shift of about 20 nm. This result supports our interpretation that the measured cut-off shift is mainly caused by fabrication-induced dimensional deviations, such as waveguide-width variation and SWG filling factor deviation.

**SUPPLEMENTARY FIGURE 2	|**	Simulated $n_{eff}$ of the isolated strip and SWG waveguides in the proposed dichroic filter, calculated using the ideal and SEM-derived geometric parameters. The red curves correspond to the ideal design parameters presented in Figure 3b, while the blue curves show the simulation results based on the device geometries extracted from the SEM images shown in Figure 4d. Solid and dashed curves represent the strip and SWG waveguides, respectively.

**7	|	Discussion of Thermo-optic Effects on Cut-off Wavelength**

The thermo-optic effect can influence the $n_{eff}$ of both waveguides in the dichroic filter, thereby modifying their dispersion curves and shifting the cut-off wavelength.

To further evaluate the possible influence of temperature, we performed an additional thermo-optic simulation of the device. Supplementary Figures 3 and 4 illustrate the thermo-optic effects on the simulated $n_{eff}$ dispersion curves of the proposed dichroic filter based on ideal device geometry and geometric parameters extracted from the SEM image shown in Figure 4d, respectively. Around 3.5 μm, the thermo-optic coefficient of silicon at 295 K was taken as approximately 1.71×10^-4^ K^-1^. Both simulations show that the cut-off wavelength shifts by approximately 3.7 nm for every 20 °C temperature change. Therefore, even a relatively large temperature variation would induce a cut-off shift much smaller than the experimentally observed ~20 nm blue shift.

Moreover, during the measurements, the experiments were carried out in a closed laboratory environment with the room temperature maintained at approximately 22 °C. The input optical power was only at the mW level and the chip was mounted on a large copper stage, which provided efficient thermal sinking. Based on this analysis, we believe that the thermo-optic effect is not the dominant origin of the measured cut-off wavelength shift. The observed shift is more likely mainly caused by fabrication-induced deviations.

After reducing systematic fabrication errors through calibrated GDS CD biasing or process compensation, temperature control could potentially be used for fine tuning of the cut-off wavelength over the last few nanometers. This will further improve the agreement between the designed and experimentally measured device performance.

**SUPPLEMENTARY FIGURE 3	|**	Simulated $n_{eff}$ of the isolated strip and SWG waveguides in the proposed dichroic filter at different temperatures, based on the ideal design parameters. The solid and dashed curves represent the strip and SWG waveguides, respectively. As the temperature decreases, the cut-off wavelength gradually shifts toward shorter wavelengths (blue-shifts).

**SUPPLEMENTARY FIGURE 4	|**	Simulated $n_{eff}$ of the isolated strip and SWG waveguides in the proposed dichroic filter at different temperatures, based on the device geometries extracted from SEM images shown in Figure 4d. The solid and dashed curves represent the strip and SWG waveguides, respectively. As the temperature decreases, the cut-off wavelength gradually shifts toward shorter wavelengths (blue-shifts).

**8	|	Interferometric Signatures in the Transmission Spectra**

The small wavelength-dependent oscillations are visible in the measured transmission spectra in Figure 5. Specifically, the ripples at the two output ports are complementary: a decrease in transmission at one port is accompanied by a corresponding increase at the other port. When the transmissions of the two output ports are converted to linear power units and summed, the resulting total transmitted power exhibits no discernible corresponding oscillations over the operating wavelength range.

These observations indicate that the oscillations mainly arise from wavelength-dependent power exchange between the two output modes, rather than from a significant variation in the total transmitted power. We attribute this behavior to a small residual non-adiabatic excitation of both supermodes in the coupling region, rather than by a strong Fabry-Pérot-type modulation of the total transmitted power. More specifically, it indicates that the geometrical variation is not sufficiently slow during the adiabatic evolution. As a result, a small fraction of the optical power can be transferred to the other supermode. The two simultaneously excited supermodes then accumulate different phases as they propagate, giving rise to wavelength-dependent modal beating and, therefore, complementary oscillations at the two output ports.

Nevertheless, the magnitude of the oscillatory power component is small. Since Figure 5 is plotted on a logarithmic scale, the oscillations appear more pronounced than they are on a linear power scale. The relevant exchanged power occurs at approximately the -15 dB level, corresponding to only about 2-3% of the input power.

The oscillations can be further suppressed by optimizing the longitudinal evolution of both the propagation-constant mismatch and the coupling strength. In particular, the strip-waveguide width and the SWG parameters can be varied more slowly near the phase-matching region, where the propagation-constant separation between the two supermodes is smallest and the adiabaticity requirement is most restricted. When the strip waveguide is gradually brought into coupling with the SWG waveguide, a nonlinear longitudinal profile incorporating a longer and more gradual transition in this critical region can improve the adiabaticity without substantially increasing the total device length. The coupling gap may also be locally reduced near the phase-matching region to increase the coupling strength and the minimum supermode separation, thereby relaxing the adiabaticity requirement.

**9	|	Insertion Losses of the Strip-to-SWG Converter**

The converter was optimized numerically to ensure adiabatic transformation between the strip waveguide mode and the targeted SWG mode, with negligible excitation of undesired higher-order modes.

Over the wavelength of 3300-3700 nm, the average simulated insertion loss is approximately -0.0047 dB, while the average measured insertion loss reaches around -0.053 dB.

**SUPPLEMENTARY FIGURE 5	|**	Simulated (red curve) and experimentally measured (blue curve) insertion losses of the strip-to-SWG converter.

**REFERENCE**

1.	Xia, L., Liu, Y., Chen, R. T., Weng, B. & Zou, Y. "Advancements in miniaturized infrared spectroscopic-based volatile organic compound sensors: A systematic review,". *Applied Physics Reviews* 11, 031306 (2024), https//doi.org/10.1063/5.0197236.

2.	Butt, M. A., Pastor Abellán, D. & Piramidowicz, R. "Mid‐Infrared Integrated Photonics: Material Platforms and Emerging Applications,". *Laser & Photonics Reviews* e00099 (2025), https//doi.org/10.1002/lpor.202500099.

3.	Zou, Y., Chakravarty, S., Chung, C.-J., Xu, X. & Chen, R. T. "Mid-infrared silicon photonic waveguides and devices [Invited],". *Photonics Research* 6, 254 (2018), https//doi.org/10.1364/PRJ.6.000254.

4.	Hu, T., Dong, B., Luo, X., et al. "Silicon photonic platforms for mid-infrared applications [Invited],". *Photonics Research* 5, 417 (2017), https//doi.org/10.1364/PRJ.5.000417.

5.	Soref, R. "Mid-infrared photonics in silicon and germanium,". *Nature Photonics* 4, 495–497 (2010), https//doi.org/10.1038/nphoton.2010.171.

6.	Vlk, M., Datta, A., Alberti, S., et al. "Extraordinary evanescent field confinement waveguide sensor for mid-infrared trace gas spectroscopy,". *Light: Science & Applications* 10, 26 (2021), https//doi.org/10.1038/s41377-021-00470-4.

7.	Luo, Z., Zhang, A., Huang, W., et al. "Aluminum Nitride Thin Film Based Reconfigurable Integrated Photonic Devices,". *IEEE Journal of Selected Topics in Quantum Electronics* 1–18 (2023), https//doi.org/10.1109/JSTQE.2023.3245290.

8.	Luque-González, J. M., Sánchez-Postigo, A., Hadij-ElHouati, A., et al. "A review of silicon subwavelength gratings: building break-through devices with anisotropic metamaterials,". *Nanophotonics* 10, 2765–2797 (2021), https//doi.org/10.1515/nanoph-2021-0110.

9.	Raguin, D. H. & Morris, G. M. "Analysis of antireflection-structured surfaces with continuous one-dimensional surface profiles,". *Applied Optics* 32, 2582 (1993), https//doi.org/10.1364/AO.32.002582.

10.	Magden, E. S., Li, N., Raval, M., et al. "Transmissive silicon photonic dichroic filters with spectrally selective waveguides,". *Nature Communications* 9, 3009 (2018), https//doi.org/10.1038/s41467-018-05287-1.

11.	Gorgulu, K. & Magden, E. S. "Ultra-Broadband Integrated Optical Filters Based on Adiabatic Optimization of Coupled Waveguides,". *Journal of Lightwave Technology* 41, 5067–5077 (2023), https//doi.org/10.1109/JLT.2023.3250944.
